# Tumor cells educate mesenchymal stromal cells to release chemoprotective and immunomodulatory factors

**DOI:** 10.1093/jmcb/mjz090

**Published:** 2019-09-03

**Authors:** Augustin Le Naour, Mélissa Prat, Benoît Thibault, Renaud Mével, Léa Lemaitre, Hélène Leray, Marie-Véronique Joubert, Kimberley Coulson, Muriel Golzio, Lise Lefevre, Eliane Mery, Alejandra Martinez, Gwénaël Ferron, Jean-Pierre Delord, Agnès Coste, Bettina Couderc

**Affiliations:** 1 Institut Claudius Regaud –IUCT Oncopole, Université de Toulouse, Toulouse, France; 2 INSERM UMR 1037, Cancer Research Center of Toulouse (CRCT), Toulouse, France; 3 UMR 152 Pharma Dev, Université de Toulouse, IRD, UPS, Toulouse, France; 4 UMR CNRS 5089, Institut de Pharmacologie et de Biologie Structurale (IPBS), Toulouse, France

**Keywords:** chemoresistance, macrophages, mesenchymal stromal cells, ovarian adenocarcinoma, chemokines

## Abstract

Factors released by surrounding cells such as cancer-associated mesenchymal stromal cells (CA-MSCs) are involved in tumor progression and chemoresistance. In this study, we characterize the mechanisms by which naïve mesenchymal stromal cells (MSCs) can acquire a CA-MSCs phenotype. Ovarian tumor cells trigger the transformation of MSCs to CA-MSCs by expressing pro-tumoral genes implicated in the chemoresistance of cancer cells, resulting in the secretion of high levels of CXC chemokine receptors 1 and 2 (CXCR1/2) ligands such as chemokine (C-X-C motif) ligand 1 (CXCL1), CXCL2, and interleukin 8 (IL-8). CXCR1/2 ligands can also inhibit the immune response against ovarian tumor cells. Indeed, through their released factors, CA-MSCs promote the differentiation of monocytes towards M2 macrophages, which favors tumor progression. When CXCR1/2 receptors are inhibited, these CA-MSC-activated macrophages lose their M2 properties and acquire an anti-tumoral phenotype. Both *ex vivo* and *in vivo*, we used a CXCR1/2 inhibitor to sensitize ovarian tumor cells to carboplatin and circumvent the pro-tumoral effects of CA-MSCs. Since high concentrations of CXCR1/2 ligands in patients’ blood are associated with chemoresistance, CXCR1/2 inhibition could be a potential therapeutic strategy to revert carboplatin resistance.

## Introduction

Chemoresistance is a major problem in the treatment of cancer. In the case of ovarian tumors, resistance can occur during treatment, or several months later, and is generally associated with a dismal prognosis. The acquired chemoresistance within the tumor cells can be caused by molecular alterations affecting metabolism, growth control and apoptosis pathways, uptake, or efflux of the drug (Lønning et al., 2013). Some chemotherapeutic agents induce a rapid host response involving a ‘storm’ of cells, cytokines, and growth factors that promote angiogenesis, tumor regrowth, metastasis, and chemoresistance (McMillin et al., 2010; Voloshin et al., 2013; Beyar-Katz et al., 2016). Thus, the microenvironment surrounding the tumor cells has been proposed to promote the acquisition of chemoresistance.

The tumor microenvironment is composed of different cell types including endothelial cells, fibroblasts, adipocytes, immune cells, and mesenchymal stromal cells (MSCs). MSCs are multipotent stromal cells that can differentiate into adipocytes, chondrocytes, osteoblasts, fibroblasts, and vascular structures (Chamberlain et al., 2007). They can be isolated from different tissues including bone marrow and adipose tissue (Phinney et al., 2007). In tumors, cancer-associated MSCs (CA-MSCs) (Shi et al., 2016) are able to stimulate tumor growth, angiogenesis, and promote chemoresistance. This phenomenon occurs through direct interactions of CA-MSCs with tumor cells (Rafii et al., 2008) and/or the release of various factors including cytokines, growth factors, exosomes, and fatty acids (Le Naour and Couderc, 2017). For example, CCL5, IL-6, and IL-8 (Wang et al., 2010, 2011; Hofer et al., 2016) have been shown to be involved in the acquisition of chemoresistance. Wang et al. (2011) have shown that autocrine IL-8 secretion by tumor cells induced their chemoresistance, while inhibiting IL-8 was able to re-sensitize the tumor cells to cisplatin and paclitaxel. These data suggest that IL-8, as well as other ligands of CXC chemokine receptors 1 and 2 (CXCR1/2) could be involved in the chemoresistance acquisition via the recruitment of MSCs around the tumor (Wang et al., 2011).

The signaling pathways activated by IL-8 (PI3K and phospholipase C) are stimulated through the interaction of the cytokine with CXCR1/2 that are expressed by neutrophils, monocytes, endothelial cells, astrocytes, microglia, and different types of tumor cells (Thomson, 1998; Hillyer et al., 2003; Ha et al., 2017). Browne et al. (2013) have reported a strong correlation between CXCR1/2 expression and the grade of ovarian tumors.

Beside its role in chemoresistance, IL-8 is a chemo-attractant for neutrophils, and may also interact with CXCR1/2 expressing monocytes (Thomson, 1998). Monocytes differentiate into macrophages when infiltrating tissues and represent an important component of the ovarian tumor microenvironment. Macrophages are particularly plastic and capable of differentiating into specific functional states in response to stromal signals. M1-macrophages have tumoricidal activity through the secretion of cytotoxic factors, while M2-macrophages generally only produce low levels of reactive nitrogen/oxygen species (ROS), exhibit low amounts of antigen-presentation, and suppress anti-tumor immunity (Mantovani et al., 2004). Several studies have evidenced the recruitment of M2-macrophages to solid tumors in response to chemotherapy (De Palma et al., 2013). These tumor-associated macrophages (TAMs) provide an immunosuppressive microenvironment (Zheng et al., 2009; Gutierrez-Gonzalez et al., 2016), participate in angiogenesis through the release of vascular endothelial growth factors and protect tumor cells against paclitaxel chemotherapy for solid tumors (Shree et al., 2011), or melphalan-induced apoptosis in the case of multiple myeloma (Beyar-Katz et al., 2016).

Overall, it has become evident that the tumor environment determines the clinical behavior of the disease, and its content has a direct impact on patients’ overall survival (Dalton et al., 2004). In the case of ovarian cancers, patients frequently develop ascites, which refers to the abnormal accumulation of fluid in the peritoneal cavity. It contains tumor cells, stromal cells, as well as the factors secreted by these different cellular populations. In an effort to study the complex interactions between the tumor microenvironment and ovarian tumor cells (OTCs), we show that naïve MSCs can acquire a CA-MSCs phenotype in proximity with OTCs, and in turn, secrete chemoprotective factors and polarize macrophages into a less cytotoxic phenotype. We then demonstrate the plasticity of this phenotype *in vivo* and *ex vivo* by re-sensitizing the tumor cells to chemotherapy using CXCR1/2 receptor inhibitors, which may be a promising therapeutic strategy to circumvent resistances in patients.

## Results

### CA-MSCs isolated from tumor biopsies confer chemoresistance to OTCs

**Figure 1:**
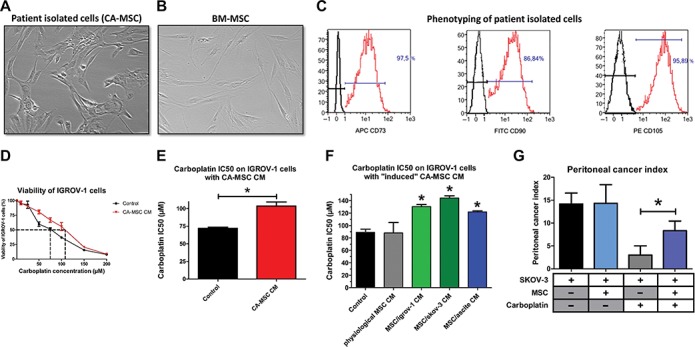
The chemoresistance acquisition by OTCs through factors secreted by CA-MSCs. (**A** and **B**) Phenotype of stromal cells from patient biopsies and BM-MSCs (X40). (**C**) Flow cytometry analysis of stromal cells from patient biopsies. The expression of CD73, CD90, and CD105 was evaluated. (**D**) OTCs cultured alone or in the presence of CA-MSC CM were treated with increasing carboplatin concentrations for 48 h. Cell viability was measured for IGROV-1 cells cultured in control medium or in CA-MSC CM. The dotted line corresponds to 50% cell viability. (**E**) Histogram representing the mean carboplatin IC50 ± SEM on IGROV-1 cells cultured with CA-MSC CM (*n* = 12). (**F**) Histogram representing the carboplatin IC50 on IGROV-1 cells cultured with BM-MSC CM (physiological MSCs) or iCA-MSC CM from different origins (BM-MSCs cultivated with IGROV-1 CM, SKOV-3 CM, or ascites) (*n* = 4 for each type of MSC). (**G**) The mean value of the peritoneal cancer index ± SEM calculated in mice is presented (*n* = 6 mice/group). *P*-values of <0.05 (*) using a Wilcoxon–Mann–Whitney test indicate a significant difference.

Isolated cells from freshly extracted human ovarian adenocarcinoma biopsies (*n* = 12) were selected based on their adherence to plastic and their fibroblast-like morphology (Figure 1A and B). These cells were CD73^+^CD90^+^CD105^+^ and presented a similar phenotype to BM-MSC (Figure 1C). According to the expression of these typical MSC markers, and the absence of CD14, CD20, CD34, and CD45 expression, we define this population as CA-MSCs.

Next, to evaluate the ability of CA-MSCs to induce chemoresistance in OTCs, we cultured the human OTC line IGROV-1 in conditioned media (CM) from CA-MSCs, and treated them with carboplatin, the standard-of-care in ovarian cancer treatment. CA-MSC CM induced an increase of 44% in the carboplatin IC50 on IGROV-1 cells (Figure 1D and E). All the CA-MSCs that we cultured (*n* = 12) were able to induce chemoresistance in IGROV-1 cells through released factors (Figure 1E; Supplementary Figure S1A). We observed a similar effect on SKOV-3 cells, with an increase of 33% in the carboplatin IC50 (Supplementary Figure S1B).

### BM-MSCs could differentiate into CA-MSCs in a tumoral microenvironment

MSCs display different phenotypes and functions, depending on the type of tissue from where they are isolated, including ovaries, bone marrow, adipose tissue, heart, and bladder (Hass et al., 2011; Stimpfel et al., 2014). Thus, we aimed to analyze whether CA-MSCs acquired specific functions in response to the surrounding OTCs. We hypothesized that CA-MSCs isolated from ovarian nodules could be MSCs pre-educated by OTCs to adopt new functions such as the ability to induce chemoresistance. To address if CA-MSCs could be differentiated cells derived from progenitor MSCs, we cultured multipotent BM-MSCs from healthy female donors either in control medium (physiological BM-MSCs), in CM obtained from two different human OTC lines (IGROV-1 or SKOV-3), or in patients ascites. MSCs cultured with human OTC lines or in patients ascite were referred to as induced CA-MSC (iCA-MSC).

While the physiological MSC CM did not confer chemoresistance to OTCs, iCA-MSC CM induced an increase in the chemoresistance of IGROV-1 cells to carboplatin (Figure 1F), similar to that observed with CA-MSC CM (Figure 1E; Supplementary Figure S1A).

In order to test our hypothesis *in vivo*, we injected intraperitoneally BM-MSCs from healthy donors to nude mice bearing ovarian tumors (SKOV-3 cells) and analyzed their ability to confer chemoresistance to OTCs. We evaluated tumor progression by measuring the peritoneal cancer index (Supplementary Table S1) as previously described (Picaud et al., 2014). The injection of BM-MSCs did not affect tumor progression (Figure 1G) but altered the efficiency of carboplatin treatment showing that BM-MSCs injected at the same time as OTCs conferred chemoresistance to OTCs *in vivo* (Figure 1G). Taken together, our results showed that BM-MSCs in the vicinity of OTCs acquired a CA-MSC phenotype, which in turn induced the acquisition of chemoresistance by OTCs*.*

### CA-MSCs and iCA-MSCs lose their multipotency

**Figure 2:**
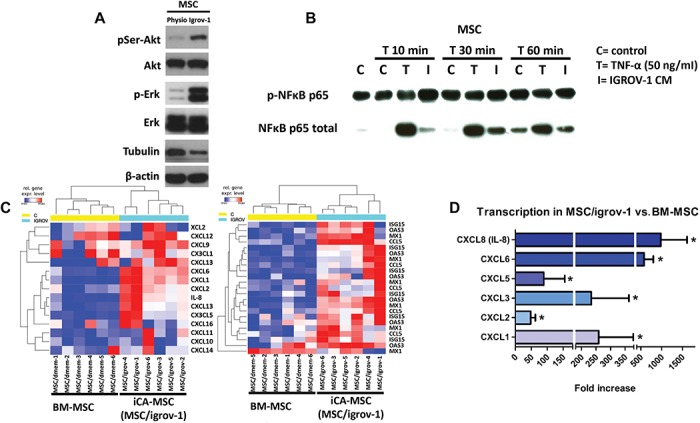
Factors secreted by OTCs activate PI3K/Akt, MAPK, and NF-κB signaling pathways and modify gene expression in MSCs. (**A**) Akt, phospho-Akt (Ser473), Erk, and Phospho-Erk expression levels were assayed by western blot on physiological BM-MSC or MSC/igrov-1 (representative of three experiments). (**B**) BM-MSCs were cultured with IGROV-1 CM or with control medium (control) for the indicated amount of time. T represents treatment of the BM-MSCs with 50 ng/ml of TNFα. NF-κB and phospho-NF-κB p65 protein expression was assayed by western blot. (**C**) Representative gene expression of physiological BM-MSCs (*n* = 6) and iCA-MSCs (MSC/igrov-1) (*n* = 6). RNA was extracted and analyzed by Nanostring™ technology. (**D**) Quantification of the transcriptomic analysis showing the log2 fold change of the selected genes with an initial filter (SD > 0.25) to eliminate genes with little variation. An ANOVA test was applied to compare the six BM-MSC control samples versus the six iCA-MSC (MSC/igrov-1) samples. The *P*-values were corrected for multiple tests using the BH method.

The activation of the three major signaling pathways involved in chemoresistance was evaluated in MSCs cultured in OTC CM. The PI3K/Akt, MAPK, and NF-κB signaling pathways were activated in iCA-MSCs derived from MSCs cultured in CM from OTC lines (IGROV-1) compared to native BM-MSCs (Figure 2A and B).

Since BM-MSCs are multipotent cells, we evaluated the capacity of CA-MSCs isolated from patient tumors to differentiate into osteoblasts or adipocytes. BM-MSC were able to differentiate into either osteoblasts or adipocytes as opposed to CA-MSCs (Supplementary Figures S2A and S3A). In contrast, BM-MSCs cultured for 21 days in control medium maintained multipotency. Similarly, BM-MSCs cultured in a tumoral environment lost their multipotency (Supplementary Figures S2B and S3B). CA-MSCs did not appear to be cancer-associated fibroblasts (CAFs) as demonstrated by the absence of upregulation of α-SMA, FAP, FSP1, or PDGFRα. On the contrary, these CAF markers were downregulated in comparison to the control BM-MSC (Supplementary Figure S4).

Overall, factors secreted by OTCs activated signaling pathways in BM-MSCs and led them to differentiate into a CA-MSCs phenotype, which is characterized by a loss of their multipotency.

### CA-MSCs and iCA-MSCs upregulate CXCL1, CXCL2, and IL-8

We observed that iCA-MSCs acquired functions such as the ability to secrete factors able to induce OTC chemoresistance. In order to determine the implicated factors, gene expression in BM-MSCs and their derived iCA-MSCs from the same donor was compared to overcome inter-individual variability. The iCA-MSCs upregulated several pro-tumoral (e.g. IL-6), pro-metastatic (e.g. CCL5), and pro-angiogenic genes (e.g. IL-8 and CCL5) (Figure 2C and D), indicating that culturing MSCs in a tumoral context is able to modify them to a pro-tumoral phenotype.

To identify the secreted factors produced by CA-MSCs and responsible for the acquisition of chemoresistance by OTCs, we analyzed data obtained from the gene expression analysis. We focused on secreted factors shown in the literature to be involved in the acquisition of chemoresistance (Castells et al., 2013). We focused on CXCR1/2 ligands, since these chemokines are known to be involved in the OTC chemoresistance to cisplatin and taxan (Wang et al., 2011) and to be associated with solid tumor progression (Ijichi et al., 2011). In particular, CXCL1 (log2 fold-change = 7.5), CXCL2 (log2 fold-change = 5.0), and IL-8 (log2 fold-change = 8.5) were found to be largely upregulated in iCA-MSCs compared to BM-MSCs (Figure 2D).

**Figure 3:**
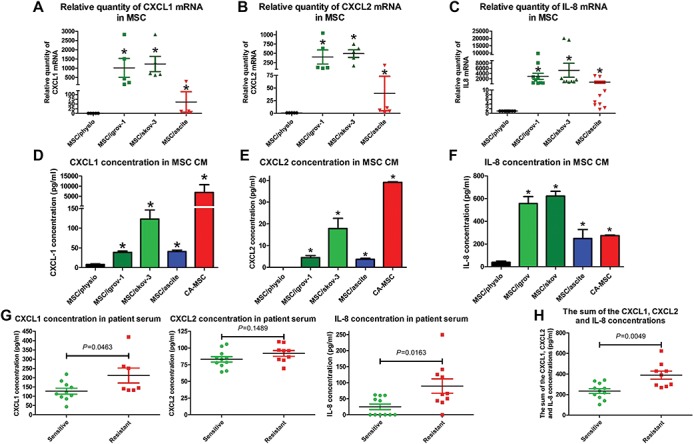
Determination of the CXCR1/2 ligands secreted by CA-MSCs and iCA-MSCs. (**A**–**C**) The upregulation of genes coding for CXCL1, CXCL2 and IL-8 was validated by RT-qPCR performed on RNA extracted from BM-MSCs and different types of iCA-MSCs (induced by IGROV-1 CM, SKOV-3 CM, or ascites). The data from BM-MSCs were set to 1 and the relative quantity of mRNA is shown. CXCL1 (**A**), CXCL2 (**B**), IL-8 (**C**). (**D**–**F**) The concentrations of CXCL1, CXCL2, and IL-8 in the CM were quantified using ELISA kits. The CM from CA-MSCs was also tested. Histograms show the mean concentrations of CXCL1 (**D**), CXCL2 (**E**), and IL-8 (**F**) from three independent experiments performed in triplicate (mean ± SEM). (**G**) The CXCL1, CXCL2, and IL-8 concentrations were determined using ELISA kits on samples of serum from patients with ovarian adenocarcinoma collected at diagnosis. (**H**) The sum of the CXCL1, CXCL2, and IL-8 concentrations was obtained by adding together the serum concentration of these three chemokines. *P*-values of <0.05 (*) using a Wilcoxon–Mann–Whitney test indicate a significant difference.

In order to confirm these results, we performed RT-qPCR and ELISA analysis on BM-MSCs or their counterpart iCA-MSCs and compared the expression of CXCL1, CXCL2, and IL-8. There was a strong upregulation of CXCL1, CXCL2, and IL-8 mRNAs and protein levels in all three types of iCA-MSCs (MSC/igrov-1, MSC/skov-3, and MSC/ascite) compared to the control condition (Figure 3A–F).

Additionally, we have evaluated whether carboplatin treatment could modify the nature of the chemokines secreted by iCA-MSC. CXCL1 secretion was enhanced by both physiological BM-MSC and iCA-MSC (Supplementary Figure S5A), while it only minimally affected CXCL2 and IL-8 production (Supplementary Figure S5B and C).

The increased chemokine secretions of iCA-MSC could be explained by the activation of the signaling pathways observed in the iCA-MSC (Figure 2A and B). However, inhibitors of PI3K, MAPK, and NF-κB (Supplementary Figure S5D–K) pathways did not completely abrogate the production of these chemokines. Therefore, we hypothesize that both MEK and NF-κB signaling pathways could be involved in the overproduction of CXCR1/2 ligands by iCA-MSCs.

Finally, we evaluated the levels of the three CXCR1/2 ligands in serum from patients with ovarian adenocarcinoma (Supplementary Table S2) collected at diagnosis. The classification of the relapse is obtained according to the duration of the platinum-free interval, corresponding to the time between the date of the last dose of platinum and the date of the relapse (Tomao et al., 2017). There was an increased concentration of the three chemokines in the serum from patients with resistant tumors compared to those with sensitive tumors (*P*-values of 0.046, 0.149, and 0.016 for CXCL1, CXCL2, and IL-8, respectively) (Figure 3G). The sum of the concentrations of CXCL1, CXCL2, and IL-8 was obtained by adding together the serum concentrations of these three chemokines, and showed that patients with resistant tumors had a higher serum concentration of these chemokines (*P* = 0.0049). Our results showed that increased levels of CXCR1/2 ligands may be predictive markers of a tumor’s response to chemotherapy. Thus, they appear as promising targets to understand mechanisms by which MSCs induce the chemoresistance acquisition in OTCs.

### CXCR1/2 inhibition reverses chemoresistance

In order to determine whether CXCR1/2 ligands could play a role in the chemoresistance mediated by MSCs, we determined the carboplatin IC50 on IGROV-1 cells in the presence of a CXCR1/2 inhibitor (Hayashi et al., 1995). We previously verified that CXCR1/2 were expressed in different human OTC lines and showed that epithelial adenocarcinoma cell lines (OVCAR-3, IGROV-1, and SKOV-3 cells) as well as a clear cell carcinoma cell line (JHOC-5) expressed both CXCR1 (Supplementary Figure S6A) and CXCR2 (Supplementary Figure S6B).

**Figure 4:**
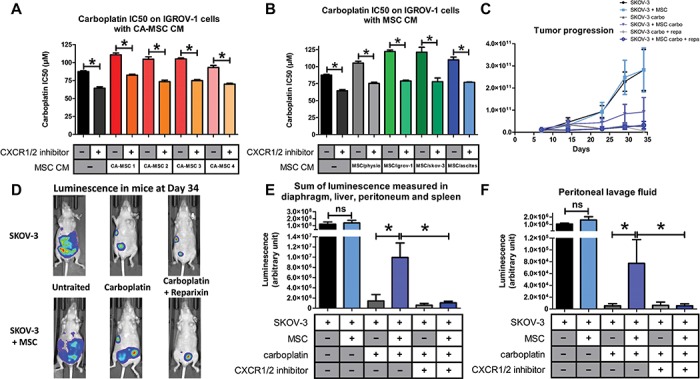
Implication of the CXCR1/2 axis in OTC resistance to carboplatin. (**A** and **B**) The carboplatin IC50 was monitored as described previously in Figure 1. IGROV-1 cells were cultured in the presence or not of CM from BM-MSCs (MSC/physio), CA-MSCs (*n* = 4), or iCA-MSCs (MSC/igrov-1 and MSC/skov-3, MSC/ascite). At Day 1, cells were treated with carboplatin admixed or not with a CXCR1/2 inhibitor (100 μM). Cell viability was evaluated at Day 3. (**C**) Bioluminescence analysis through the whole body of the mice was performed once a week after an intraperitoneal injection of luciferin. (**D**) At Day 34, the whole-body bioluminescence of the mice was analyzed making it possible to obtain photographs to visualize and to quantify the luminescence illustrated here using one mouse per group. (**E** and **F**) On Day 36, the mice were euthanized and a peritoneal lavage was carried out with 5 ml of NaCl 0.9%. The peritoneum, spleen, liver, and diaphragm were removed. After addition of luciferin, the sum of the luminescence of the peritoneum, spleen, diaphragm, and liver (**E**) and the luminescence in the peritoneal lavage fluid (**F**) was measured. *P*-values of <0.05 (*) using a Wilcoxon–Mann–Whitney test indicate a significant difference.

While the CXCR1/2 inhibitor alone did not alter the viability of OTCs (Supplementary Figures S6C, S7A and B), it induced an increase in their sensitivity to carboplatin when cultured in control medium. This is due to the inhibition of the autocrine production of IL-8 by the tumor cells (Pasquet et al., 2010). The acquired chemoresistance by IGROV-1 cells mediated through factors secreted by CA-MSCs or iCA-MSC could be reversed by the CXCR1/2 inhibitor (Figure 4A and B).

To confirm that CXCR1/2 inhibition could re-sensitize OTCs, we used our *in vivo* murine model with the CXCR1/2 inhibitor reparixin (Kim et al., 2011). The co-administration of MSCs with OTCs did not affect tumor development (Figure 4C). Carboplatin treatment effectively reduced tumor development in mice, but significantly less when OTCs where co-injected with MSCs, confirming that MSCs induced chemoresistance to carboplatin. Interestingly, CXCR1/2 inhibition reversed the chemoresistance induced by MSCs, as tumor progression was abolished when mice were treated with a mix of carboplatin and CXCR1/2 inhibitor (reparixin) (Figure 4D and E). Therefore, we conclude that CXCR1/2 inhibitor can reverse the acquired chemoresistance mediated by MSCs via CXCR1/2 ligands.

### MSCs could play a role in the anti-tumoral activity of immune cells

**Figure 5:**
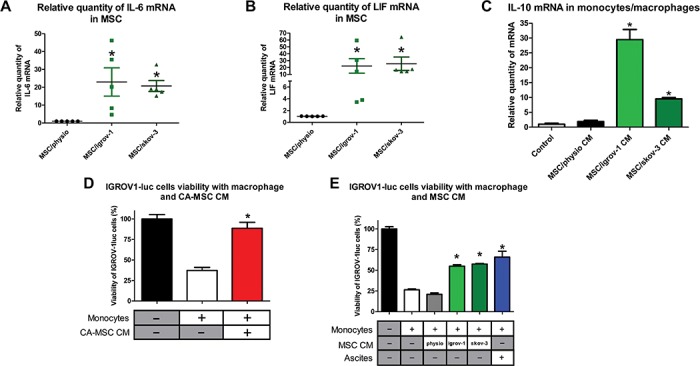
CA-MSCs facilitate monocyte to macrophage differentiation towards the M2 TAM phenotype. (**A** and **B**) The relative expression of mRNA coding for IL-6 (**A**) and LIF (**B**) was evaluated on BM-MSCs and the iCA-MSCs (induced by IGROV-1 (MSC/igrov-1) or SKOV-3 (MSC/skov-3) CM). The data from BM-MSCs were set to 1 and the relative quantity of mRNA is shown. (**C**) IL-10 mRNA expression levels were analyzed in human monocytes cultured in control media or in MSC/physio, MSC/igrov-1, or MSC/skov-3 CMs. The data from control media were set at 1 and the relative quantity of mRNA is shown. (**D** and **E**) The cytotoxic activity of the macrophages that have been cultured in different media (CA-MSC, BM-MSC, MSC/igrov-1, or MSC/skov-3 CMs or ascites) toward the IGROV-1luc (expressing luciferase) cells was evaluated by measuring luciferase activity. Bar graphs representing the IGROV-1luc cells viability (%) (*n* = 4). *P*-values of <0.05 (*) using a Wilcoxon–Mann–Whitney test indicate a significant difference.

We and others have shown that factors secreted by MSCs are involved in re-educating macrophages by manipulating metabolic programs in differentially polarized macrophages (Castells et al., 2012; Vasandan et al., 2016) such as IL-8 (Gerszten et al., 1999), IL-6, and LIF (Duluc et al., 2007; Castells et al., 2012). Transcriptomic analysis (Figure 2A) revealed that iCA-MSCs upregulated factors involved in the activation of immune cells, in particular CXCR1/2 ligands, IL-6, and LIF (Figure 5A and B), as well as CCL5, CXCL3, CXCL5, and CXCL6.

To clarify which factors secreted by MSCs are involved in macrophage polarization, we cultured monocytes from healthy donors in the presence of CM from BM-MSCs (physiological), CA-MSCs, or iCA-MSCs. The expression levels of M2-specific genes (Figure 5C; Supplementary Table S3) were increased in the monocytes/macrophages cultured in the presence of CM from iCA-MSCs. Therefore, iCA-MSC CM were able to induce the upregulation of M2-specific markers, suggesting a conversion of naive monocytes into M2 macrophages.

M2 macrophages are generally pro-tumoral, and they do not have the tumoricidal properties of M1 macrophages. In order to assess this phenotype, we cultured luciferase expressing IGROV-1 (IGROV1-luc) with monocytes cultured in control medium, in CM from CA-MSCs (Figure 5D) or iCA-MSCs (MSC/igrov-1 or MSC/skov-3), or in ascites. Monocytes were cultured in ascites as a positive control for the M2 polarization of naïve monocytes, as described by Duluc et al. (2007). In co-cultured conditions with either control medium or in BM-MSC CM, the monocytes were able to kill 70%–80% of the OTCs. On the contrary, when naïve monocytes were cultured in CM from CA-MSCs or iCA-MSCs, they lost their ability to kill OTCs, and instead enhanced their proliferation (Figure 5D and E).

In addition to their role in promoting the chemoresistance of OTCs, we conclude that CA-MSCs can be involved in polarizing macrophages via abolition of their tumoricidal functions.

### CXCR1/2 inhibition restores the tumoricidal function of macrophages

**Figure 6:**
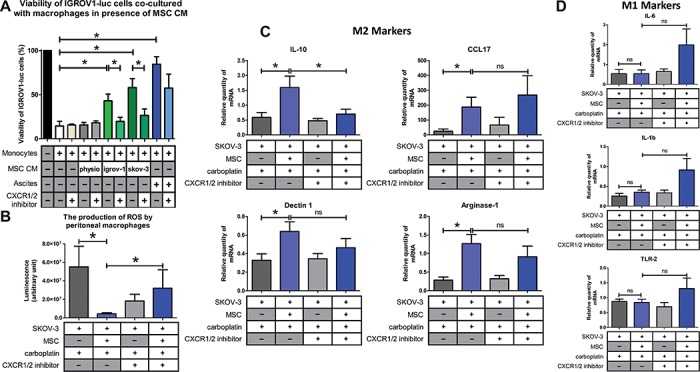
CXCR1/2 inhibition prevented the M2 macrophage polarization induced by CA-MSCs. (**A**) The cytotoxic activity of the macrophages was evaluated as described Figure 5D and E in the presence of a CXCR1/2 inhibitor. (**B**) The ROS production by peritoneal macrophages isolated from mice injected with SKOV-3 cells admixed or not with MSCs and treated or not with carboplatin +/− CXCR1/2 inhibitor was analyzed. (**C** and **D**) Gene expression analysis of peritoneal macrophages was analyzed by RT-qPCR (8 mice/group) on M2 (IL-10, CCL17, dectin-1, and arginase-1) and M1 (IL-6, IL-1β, and TLR-2) markers. *P*-values of <0.05 (*) using a Wilcoxon–Mann–Whitney test indicate a significant difference.

Since CA-MSC and iCA-MSC induced a modification of the macrophage tumoricidal activity, and because this observation could be associated with a difference in CXCL1/2 and IL-8 secretions between these cells, we evaluated the impact of CXCR1/2 inhibition on the ability of macrophages cultured in CA-MSC CM to kill OTCs. We cultured IGROV1-luc with naïve monocytes either in control medium, in CM from CA-MSCs or iCA-MSCs (MSC/igrov-1 and MSC/skov-3), or in ascites, in the presence or absence of the CXCR1/2 inhibitor. While naïve monocytes cultured in CM from CA-MSCs or iCA-MSCs or in ascites were not able to efficiently kill OTCs, the same monocytes had greater tumoricidal ability when cultured in the same conditions in the presence of the CXCR1/2 inhibitor (Figure 6A). The CXCR1/2 inhibitor effect was particularly more pronounced on the monocytes cultured in CM from CA-MSCs than on the monocytes cultured in ascites.

Since the CXCR1/2 inhibitor restored the anti-tumoral properties of macrophages *in vitro*, we wondered if it would be similar *in vivo*. M1 macrophages are characterized by high levels of ROS production (Tan et al., 2016). We therefore measured ROS production in macrophages isolated from mice that were injected with OTCs with or without MSCs and treated or not with a combination of carboplatin and the CXCR1/2 inhibitor (reparixin). We observed that the presence of MSCs caused a decrease in the ROS production by peritoneal macrophages. When mice injected with OTCs and MSCs were treated with reparixin, we observed an increase in the ROS production of peritoneal macrophages (Figure 6B) suggesting a polarization of the macrophages to an M1 phenotype. This observation could explain the slower tumor progression observed in this condition (Figure 4C). In addition to the ROS production, part of the tumoricidal effect of peritoneal macrophages observed in mice could be due to an increase in phagocytosis observed in macrophages from mice injected with MSCs and treated with carboplatin and reparixin (Supplementary Figure S7C).

When mice were injected with a combination of SKOV-3 cells and MSCs, and then treated with carboplatin, there was a high proportion of IL-10, arginase-1, dectin-1, and CCL17-positive macrophages, suggesting a M2 polarization compared to the mice injected with SKOV-3 cells alone (Figure 6C). When mice were co-treated with carboplatin and reparixin, macrophages did not express as much IL-10 levels, suggesting a shift in macrophage polarization. With regards to the M1 markers (Mantovani et al., 2004), our data suggest a slight upregulation of IL-1β, IL-6, and TLR-2 expression in the presence of MSCs and reparixin (Figure 6D), despite the fact that gene expression profiles reveal usually an M1/M2 mixed-polarization phenotype in ovarian cancer (Reinartz et al., 2014; Zheng et al., 2017). The effect of reparixin appeared to be dependent on CXCR1/2 ligands secretions by MSCs because the CXCR1/2 inhibitor had no effect on the macrophage polarization in absence of these cells (Supplementary Figure S7D and E).

In order to evaluate whether the chemoresistance observed in the presence of MSCs was due to differential macrophage polarization, we performed an *in vivo* experiment to deplete macrophages in mice by clodronate injection. Upon macrophage depletion, the protective nature of MSCs was no longer effective (Supplementary Figure S7A and B). Similarly, in this context, the use of reparixin had no additive antitumor effect when combined with carboplatin (Supplementary Figure S7A and B). Thus, most of the chemoprotective effect of MSCs observed *in vivo* appears to be a consequence of macrophage polarization.

**Figure 7:**
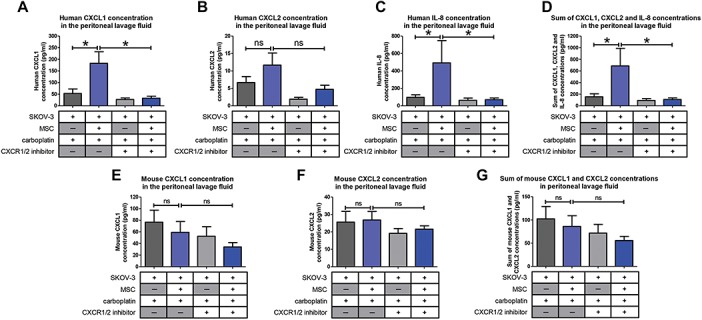
Levels of pro-inflammatory cytokines in the mouse peritonea. Levels of human and mouse chemokines CXCL1, CXCL2, and IL-8 in the mice peritonea were evaluated by ELISA (8 mice/group). *P*-values of <0.05 (*) using a Wilcoxon–Mann–Whitney test indicate a significant difference.

In line with our previous results, we observed an increase in the concentration of human CXCL1, CXCL2, and IL-8 (Figure 7A–C, respectively) in the peritonea of mice injected with SKOV-3 cells and MSCs. When SKOV-3 and MSC tumor-bearing mice were treated with reparixin, we observed a decrease in the amount of the three cytokines, suggesting that a blockade of the CXCR1/2 leads to a reduction in the expression of these chemokines (Figure 7D). Of note, the endogeneous mouse CXCL1 and CXCL2 levels were not affected (Figure 7E–G). We propose that decreasing number of OTCs could reduce the recruitment of MSCs to peritoneal tumors and their differentiation in CA-MSC, therefore resulting in a reduced concentration of peritoneal chemokines.

**Figure 8:**
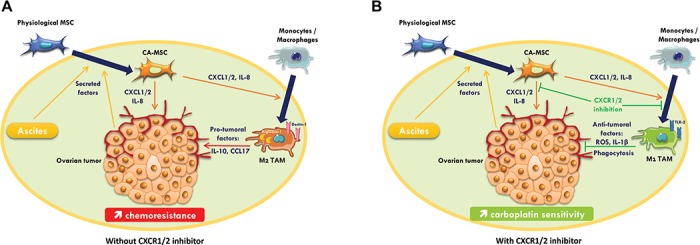
Roles of MSCs and macrophages on ovarian tumor. (**A**) Secreted factors by OTCs induce differentiation of MSC into CA-MSC, which in turn lead to the secretion of chemopreventive factors such as CXCL1/2 and IL-8. These chemokines polarize monocytes/macrophages to a M2 pro-tumoral phenotype promoting tumor growth. (**B**) CXCR1/2 inhibition restores carboplatin sensitivity in OTCs and reinstates the anti-tumoral activity of tumor-associated macrophages, thereby improving treatment efficiency.

Altogether, our results show that through their released factors, CA-MSCs can protect OTCs to carboplatin but can also trigger monocyte differentiation to a pro-tumoral M2 phenotype, favoring tumor progression and the acquisition of chemoresistance by OTCs. When CXCR1/2 are inhibited, these CA-MSC-activated macrophages lose their M2 phenotype and exhibit anti-tumoral functions. Inhibition of CXCR1/2 is able to counteract the pro-tumoral effect of the microenvironment by sensitizing OTCs to carboplatin and inducing anti-tumor immunity (Figure 8).

## Discussion

In this study, we showed that MSCs recruited to an injury site such as a cancer nodule (CA-MSCs) presented specific properties compared to naïve BM-MSCs. We showed that CA-MSCs did not influence the OTC dissemination or proliferation either *in vitro* or *in vivo,* but instead played a role in the acquired chemoresistance of OTCs against carboplatin. In addition, we demonstrated that CA-MSCs are involved in the recruitment and the polarization of macrophages. We characterized the precise mechanisms by which CA-MSCs exert their biological functions, in particular via secreting CXCR1/2 ligands. We studied the role played by these ligands, while other authors have mainly focused their attentions on IL-6 and its role in cancer cell proliferation (Wang et al., 2010; Lo et al., 2011; Qiao et al., 2018).

CA-MSCs may differentiate from MSCs of different origins. In this study, we obtained iCA-MSCs by culturing BM-MSCs from healthy female donors at 60–70 years of age in the presence of CM from OTCs or in the presence of ascites. We were able to transform BM-MSCs to CA-MSCs via the secreted factors present in their microenvironment. Interestingly, ascitic fluid from cancer patients had more or less the same effect on the differentiation of BM-MSCs. These data indicated that factors secreted by OTCs are sufficient to induce the chemoprotective phenotype of CA-MSCs, and that ascites contain factors secreted by various cell types resulting in a comparable phenotype. This work is supported by the recent published data by Coffman et al. (2019) who showed that ovarian CA-MSCs are reprogrammed in the vicinity of the tumor.

We found that BM-MSCs cultured in IGROV-1 CM (iCA-MSCs) lost their ability to differentiate into osteoblasts and adipocytes and behaved as CA-MSCs isolated from tumor biopsies of ovarian adenocarcinomas. We have shown that through their secreted factors, cancer cells can educate MSCs depending on the tumor microenvironment, therefore validating the hypothesis that tumor cells can influence the function of MSCs. However, it remains to be determined to what cellular state these CA-MSCs would have differentiated into, and what markers and function would define them. While it may be plausible to find them differentiating into CAF, we were not able to characterize them by the expression of classical markers such as αSMA, PDGFR, FSP1, or FAP.

Even if iCA-MSCs obtained from BM-MSCs adopted a phenotype similar to the CA-MSCs found in ovarian adenocarcinomas, it does not prove that CA-MSCs originate from BM-MSCs. These CA-MSCs could originate from MSCs recruited to the tumor site. Nevertheless, they could also derive from ‘resident’ MSCs located in the tissues where the tumor develops. Indeed, MSCs are not restricted to the bone marrow and are found in virtually all tissues, including ovaries (Stimpfel et al., 2014). Another possible origin could be the adipose tissue, a source of MSCs called adipose tissue-derived stromal cells (ADSC). Thus, especially during peritoneal carcinomatosis, ADSCs from the epiploon could constitute a source of CA-MSCs, given their proximity to the OTCs disseminated in the peritoneum.

CXCL1, CXCL2, CXCL3, CXCL5, CXCL6, CXCL8 (IL-8), and CCL5 were found to be upregulated in iCA-MSCs. As CXCL1, CXCL2, and IL-8 interact with the same receptors (CXCR1/2), we analyzed their combined interactions and implications for the acquisition of chemoresistance and the recruitment of immune cells in our model. We confirmed that their upregulation was correlated to patient prognosis. We found that the serum concentrations of these three chemokines, measured in ovarian adenocarcinoma patients at the time of diagnosis, predicted the sensitivity profile of the patients to platinum-based chemotherapy. To corroborate this retrospective study, it would be of interest to perform a prospective study to show that chemokine assays can predict patient treatment response, in order to adjust the treatment regimen.

We found IL-8 to be the most highly upregulated cytokine in CA-MSCs or iCA-MSCs. It has been reported to promote angiogenesis and cancer growth (Wang et al., 2015), therefore in previous experiments, we tried to abolish the chemoresistance acquisition by OTCs using an antibody directed against IL-8 (Skov et al., 2008). We observed that the antibody had only a very weak effect, suggesting that other molecules were playing an important role, and/or that the pathway involving IL-8 receptors (CXCR1/2) was not inhibited because other molecules such as CXCL1 and CXCL2 could activate these receptors.

Hence, in this study, we analyzed the effect of the inhibition of the IL-8 receptors, CXCR1/2. Reparixin, a CXCR1/2 inhibitor, had previously been used in a mouse model of pancreatic ductal adenocarcinoma to disrupt tumor/fibroblast interactions and improve overall survival in mice (Ijichi et al., 2011). We found that the CXCR1/2 inhibitor could revert the acquired chemoresistance of OTCs both *in vitro* and *in vivo*. Therefore, reparixin is an effective drug to prevent acquired carboplatin chemoresistance. Reparixin is currently being tested in patients with metastatic non-human epidermal growth factor receptor (HER2)-amplified breast cancers in an open label Phase 1b clinical study (REP0111) in combination with paclitaxel. This study has demonstrated the safety and tolerability of the combination and recorded objective responses. A 30% response rate was recorded, with durable responses of longer than 12 months in two patients (Schott et al., 2017) (https://clinicaltrials.gov/ct2/show/NCT02370238).

In the case of cancer, IL-8 (as well as CXCL1 or CXCL2) is known to be involved in angiogenesis and the recruitment and activation of immune cells. Castells et al. (2012) observed increased recruitment of macrophages to the tumor site in the presence of CA-MSCs. In fact, monocytes, upon sensing several environmental stresses are recruited to damaged and infected tissues as well as tumor sites and differentiate to macrophages en-route (Vasandan et al., 2016). Previous work performed in our laboratory suggests that CA-MSCs may be able to influence the phenotype of peritoneal macrophages by polarizing them to a pro-tumoral phenotype (M2) (Castells et al., 2012). In addition, in the case of inflammation, MSCs can induce distinct alterations in human macrophage polarization programs depending on the activation module at macrophage interface (Vasandan et al., 2016). In hematological diseases such as multiple myeloma, MSCs and macrophages can interact to induce a distinct state of macrophage polarization (Kim et al., 2012; Asimakopoulos et al., 2013). IL-8, known to induce the chemotaxis of immune cells to the tumor site, may play a role in CA-MSC-induced macrophage recruitment. It could also be the factor responsible for the CA-MSC-induced polarization of macrophages to a pro-tumoral phenotype. Dijkgraaf et al. (2013) have shown that carboplatin chemotherapy increases the number of cancer-supporting M2 macrophages. Our experimental model allowed us to isolate peritoneal macrophages and determine that MSCs could induce M2 polarization. Co-administration of reparixin and carboplatin was able to decrease the transcription of IL-10, a major marker of M2-type macrophages. This combined treatment also led to an increase of ROS production by peritoneal macrophages and an upregulation of the M1 markers: IL-1β, IL-6, and TLR-2. Therefore, reparixin can sensitize OTCs to carboplatin by decreasing the proportion of M2 macrophages or by repolarizing these macrophages to an anti-tumoral M1 phenotype.

Finally, when SKOV-3 + MSC tumor-bearing mice were treated with reparixin, we observed a decrease in the levels of peritoneal CXCR1/2 ligands, suggesting that a CXCR1/2 blockade leads to a decrease in the expression of its ligands. In addition, we propose that a decrease in the number of tumor cells could reduce the number of MSCs recruited to peritoneal tumors and differentiated in CA-MSC, resulting in a decreased concentration of the chemokines in the peritoneum. Therefore, CXCR1/2 inhibition could have a direct effect on macrophages by restoring their anti-tumoral activity. Moreover, it could also have an indirect effect by decreasing the levels of chemokines secreted by CA-MSCs, which would reduce M2 macrophage polarization.

To conclude, we showed that CA-MSCs, which are part of the ovarian tumor microenvironment, can induce OTCs resistance to chemotherapy such as carboplatin. These CA-MSCs secrete chemokines, including IL-8, CXCL1, and CXCL2 that bind to CXCR1/2. The combination of a CXCR1/2 inhibitor and platinum-based chemotherapy may be a useful strategy to restore carboplatin sensitivity in OTCs. This strategy may also modify the phenotype of tumour-associated macrophages and reinstate their anti-tumoral activity.

## Materials and methods

### Cell culture

The human ovarian adenocarcinoma IGROV-1 (a gift from the Gustave Roussy Institute, Paris) and SKOV-3 (ATCC: HTB-77) cell lines were grown in RPMI supplemented with fetal calf serum (10%), L-glutamine (1%), and penicillin/streptomycin (1%).

Primary BM-MSCs from donors that had undergone orthopedic surgery (Médipole Clinic - Toulouse), CA-MSCs from ovarian cancer patients, were grown in DMEM supplemented with fetal calf serum (10%), L-Glutamine (1%), and penicillin/streptomycin (1%).

Primary human macrophages derived from peripheral blood mononuclear cells (PBMC) were cultured in the Macrophage-SFM medium™ (Gibco).

All cells were cultured at 37°C in 5% CO_2_. They were regularly treated with Normocin™ (Invivogen) (100 μg/ml), to prevent mycoplasma contamination.

### Ascites and CM

Ascite samples (*n* = 10) from ovarian cancer patients were obtained from the biological resource center bank at the IUCT-Oncopole. The ascitic fluid was centrifuged at 300 *g* for 5 min. Supernatants were pooled and filtered at 0.2 μm. The CM from BM-MSCs, CA-MSCs, IGROV-1, and SKOV-3 cells corresponded to the supernatant of the cell culture media from confluent cells after 3 days of growth and filtered at 0.2 μm.

### Isolation of CA-MSCs from patient biopsies

From fresh tumor biopsies of patients with ovarian cancer, cells were isolated according to their plastic adhesion and then sorted by FACS using the MSC Phenotyping Kit, human (Miltenyi Biotec). CD73^+^ CD90^+^ CD105^+^ and CD14^−^ CD20^−^ CD34^−^ CD45^−^ cells were considered to be CA-MSCs.

### Generation of iCA-MSCs

BM-MSCs were cultured in DMEM diluted 1:1 either with CM from the different types of OTCs or ascites. The medium was renewed twice a week. iCA-MSCs were generated after 21 days. Three types of iCA-MSCs, MSC/igrov-1, MSC/skov-3, and MSC/ascite, were induced using the CM of IGROV-1 or SKOV-3 cells or ascites, respectively. To generate CM from iCA-MSCs, media were replaced by complete DMEM and 3 days later, supernatants were filtered at 0.2 μm. MSCs cultured in complete DMEM were named physiological BM-MSCs. After 21 days in different CM, MSCs were treated with carboplatin (50 μM, Fresenius Kabi), the pan-PI3K inhibitor GDC0941 (1 μM, Axon), the MEK inhibitor AZD6244 (1 μM, Apexbio), the NF-κB inhibitor Bay 11-7082 (10 μM, Sigma-Aldrich), or vehicle (DMSO, 0.05%, Sigma-Aldrich) in complete DMEM. Three days later, supernatants were filtered at 0.2 μM.

### Cell viability tests

IGROV-1 cells (5×10^3^) were added per well into 96-well plates in the presence of CM from the different types of MSCs (described above) diluted 1:1 in complete RPMI. After 24 h, cells were treated with a range of concentrations of carboplatin (Fresenius Kabi) (15.625–1000 μM) with or without the CXCR1/2 inhibitor (AS-62401, AnaSpec, 100 μM). After 48 h of treatment, cell viability was evaluated using the WST-8 Cell Counting Kit (Dojindo) according to the manufacturer’s instructions.

### OTC viability in co-culture with macrophages

PBMC-derived monocytes (5×10^4^) were added per well into 96-well white plates and were cultured in CM (diluted 1:1 with Macrophage-SFM (Gibco™)) from BM-MSCs, CA-MSCs, iCA-MSCs or in ascites. After 24 h, 2.5×10^4^ IGROV-1luc were added into the wells. After 3 days of co-culture, IGROV-1luc cells viability was evaluated by bioluminescence using the Steady-Glo® Luciferase Assay System (Promega) according to the manufacturer’s instructions.

### Western blot analysis

Protein extractions were performed and 15 μg of extracted proteins were separated by SDS–PAGE and revealed by antibodies directed against actin (1:1000, Cell Signaling Technology, #8457), PDGFRα (1:1000; Cell Signaling Technology, #3174), and α-smooth muscle actin (1:1000, Cell Signaling Technology, #19245).

### RNA extractions

RNA was extracted from MSCs using the RNAprotect Cell Reagent and the RNeasy Plus mini kit (QIAGEN) according to the manufacturer’s instructions. RNA was extracted from murine macrophages after 2 h of adhesion in 48-well plates, using the RNAqueous-Micro Total RNA Isolation kit (Thermo Fisher Scientific) according to the manufacturer’s instructions.

### Transcriptomic analysis by Nanostring Technology

The total RNAs extracted from BM-MSCs and iCA-MSCs (six sample pairs) were analyzed by Nanostring® technology with the ‘nCounter PanCancer Immune Profiling Panel’ to study the transcription of 770 genes (Cesano, 2015). A selection of 29 housekeeping genes was used to calculate the relative amount of target RNA.

### Transcriptional analysis by RT-qPCR

Complementary DNA (cDNA) derived from total RNA was synthesized using the Verso cDNA Synthesis Kit (Thermo Fisher Scientific), according to the manufacturer’s instructions. PCR was performed using the LightCycler® 480 SYBR Green I Master (Roche). The primers used are listed in the Supplementary Table S4.

### Quantification of chemokine concentrations by ELISA

The concentrations of murine and human CXCL1, murine and human CXCL2, and human IL-8 were determined by ELISA using the respective ELISA kits, EK0722, EK0723, EK0452 (Boster Biological Technology), ARG80185 (Arigo biolaboratories), EK0413 (Boster Biological Technology), and the IL-8 DuoSet® ELISA Development System (R&D Systems), according to the manufacturer’s instructions.

### Transcriptional analysis to study macrophage polarization

PBMC (1×10^5^) were added per well in 48-well plate wells. Macrophages were selected by adhesion (2 h). Then, they were immediately brought into contact with CM from BM-MSCs or iCA-MSCs. Twenty-four hours later, macrophage RNAs were extracted as previously described.

### Animals

The 4 to 5-week-old female Swiss nude athymic mice (Charles River laboratories, France) were housed according to the standards of the Federation of European Laboratory Animal Science Associations. They were included in experiments after one week of quarantine.

### *In vivo* experiments

SKOV-3 or SKOV-3luc cells (1×10^7^) with or without 10^6^ MSCs were injected intraperitoneally into nude mice. Treatments began at Day 7 and consisted of carboplatin injection (200 μl/mouse of a solution of 4.5 mg/ml diluted in 0.9% NaCl, Fresenius Kabi) every 7 days for 3 weeks and reparixin injection (30 mg/kg diluted in a solution of DMSO/PBS (*v*/*v*), AdooQ Bioscience) three times a week for 3 weeks. Intraperitoneal injection of clodronate liposome (200 μl/mouse of a solution of 1.25 mg/ml diluted in PBS, CliniSciences, #16001003) began one day prior cell injection and then once a week for 4 weeks. Tumor progression was monitored after 4 weeks by determining the peritoneal cancer index as described in Supplementary Table S1.

### Bioluminescence

Mice were anesthetized by inhalation of isoflurane (Abbott) at 3% with 1 L/min flow of oxygen. An injection of luciferin (XenoLight D-Luciferin-K + Salt Bioluminescent Substrate, PerkinElmer) was performed intraperitoneally in each mouse 10 min prior to analysis with IVIS® Spectrum *in vivo* imaging system (PerkinElmer), according to the manufacturer’s instructions. Normal mouse behaviour was evaluated before returning them to their original housing. Images were analyzed by Living Image Software (PerkinElmer) for evaluation and quantification.

### Organ luminescence

After euthanasia, the peritonea, spleens, livers, and diaphragms of the mice were removed. These organs were dissected and placed in 96-well white plates. The peritoneal lavage fluid (150 μl) was also placed in white plates. After the addition of luciferin (150 μg/ml, XenoLight D-Luciferin-K + Salt Bioluminescent Substrate, PerkinElmer), organ luminescence was measured using the EnVision™ Multilabel Plate Reader (PerkinElmer).

### Isolation of peritoneal macrophages

The peritoneal lavage fluid (see above) was centrifuged (300 *g* for 10 min). After lysis of the red blood cells, cells were separated using percoll (GE Healthcare Life Sciences, 17-0891), according to the manufacturer’s instructions. Cells (1×10^5^) from the macrophage-containing fraction were inoculated in 48-well plates. The macrophages were selected by adhesion (2 h). ROS production, as well as their mRNA expression levels were evaluated as described below or previously, respectively.

### ROS production

After 2 h of adhesion at 37°C and 5% CO_2_, the NADPH oxidase activity of 10^5^ peritoneal macrophages was measured by chemiluminescence in the presence of 60 μM of a chemiluminogenic probe (5-amino-2,3-dihydro-1,4-phthalazinedione, Sigma-Aldrich). Chemiluminescence production was analyzed continuously for 90 min with a luminometer (EnVision™ Multilabel Plate Readers, PerkinElmer).

### Macrophage phagocytosis assay

Peritoneal macrophages were co-cultured 2 h at 37°C with pHrodo™ Red Zymosan Bioparticles™ Conjugate for Phagocytosis (Thermofischer Scientific), according to the manufacturer’s instructions. Fluorescence was measured with a plate reader luminometer (Envision, PerkinElmer).

### Statistics

For chemoresistance tests, RT-qPCRs, co-cultures, cytometry data, and *in vivo* test results, the comparison between groups was performed using a Wilcoxon–Mann–Whitney test (independent non-parametric data). *P*-values < 0.05 indicate a significant difference. An ANOVA test was applied to compare the six BM-MSC control samples versus the six iCA-MSC (MSC/igrov-1) samples. The *P*-values were corrected for multiple tests using the Benjamini and Hochberg (BH) method.

### Study approval

All experiments involving animals were performed in accordance with the relevant European guidelines and regulations. The protocols and the experiments were approved by the Claudius Regaud Institute animal ethics committee (approval number: ICR-2015-06).

Human studies: All human biopsies and sera came from patients who provided written informed consent prior to inclusion in the study. The study has been approved by the IUCT-O (Toulouse University Institute of Cancer-Oncopole) ethics committee.

## Supplementary Material

JMCB_2019_0006_Supplementary_Material_mjz090
