# Supplementary material for: Tumor cells educate mesenchymal stromal cells to release chemoprotective and immunomodulatory factors
Source: J Mol Cell Biol. 2019 Sep 3;12(3):202–15. doi: 10.1093/jmcb/mjz090 (PMC7181721; doi:10.1093/jmcb/mjz090)
Supplement: JMCB_2019_0006_Supplementary_Material_mjz090 [file jmcb_2019_0006_supplementary_material_mjz090.pdf]

Supplementary material

Tumor cells educate mesenchymal stromal cells to release chemoprotective and immunomodulatory factors

Augustin Le Naour[1,2]#, Mélissa Prat[3]#, Benoît Thibault[1,2], Renaud Mével[1,2], Léa Lemaitre[1,2], Hélène Leray[1,2], Marie-Véronique Joubert[1,2], Muriel Golzio[4], Lise Lefevre[3], Eliane Mery[1], Alejandra Martinez[1], Gwénaël Ferron[1], Jean-Pierre Delord[1,2], Agnès Coste[3]¶, Bettina Couderc[1,2]¶†.

1: Institut Claudius Regaud –IUCT Oncopole, Université de Toulouse, Toulouse, France

2 : INSERM UMR.1037-Cancer Research Center of Toulouse (CRCT), Toulouse, France

3: UMR 152 Pharma Dev, Université de Toulouse, IRD, UPS, Toulouse, France,

4: UMR CNRS 5089, IPBS Toulouse, France

*Correspondence should be addressed to: Couderc.bettina@iuct-oncopole.fr

Content

I. Supplementary figures p. 3

Supplementary Figure S1: The chemoresistance acquisition by SKOV-3 and IGROV-1 cells through factors secreted by CA-MSCs p. 3

Supplementary Figure S2: Functional characterization of CA-MCS multipotency. p. 4

Supplementary Figure S3: Characterization of CA-MCS multipotency by transcriptional analysis. p. 5

Supplementary Figure S4: Test of iCA-MSC differentiation in CAF p. 6

Supplementary Figure S5: Effects of treatments on the chemokine production by MSCs p. 7

Supplementary Figure S6: Expression of CXCR1/2 and sensitivity to its inhibition in OTCs p. 8

Supplementary Figure S7: Effects of the CXCR1/2 inhibitor are lost in the absence of macrophages and require their prior polarization by MSCs p. 9

II. Supplementary tables p. 10

Supplementary Table 1: Peritoneal cancer index p. 10

Supplementary Table 2: Baseline characteristics of patient samples. p. 11

Supplementary Table 3: Effect of iCA-MSC secretions on the transcription of M2 polarization marker genes in primary human monocytes p. 12

Supplementary Table 4: Primers used for qRT-PCR analysis p. 13

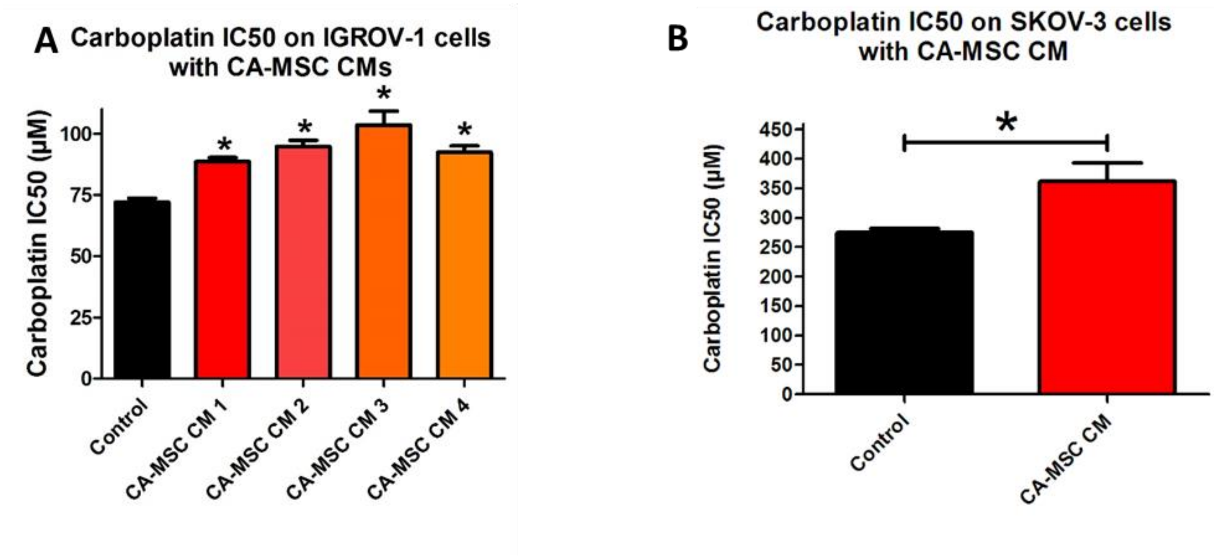

Supplementary Figure S1: The chemoresistance acquisition by SKOV-3 and IGROV-1 cells through factors secreted by CA-MSCs

A: Histogram representing the mean IC50 of carboplatin on SKOV-3 cells cultured with CA-MSC CM (n=6). B: Histogram representing the mean IC50 of carboplatin on IGROV-1 cells cultured with CA-MSC CM from 4 different patients (each tested in triplicate).

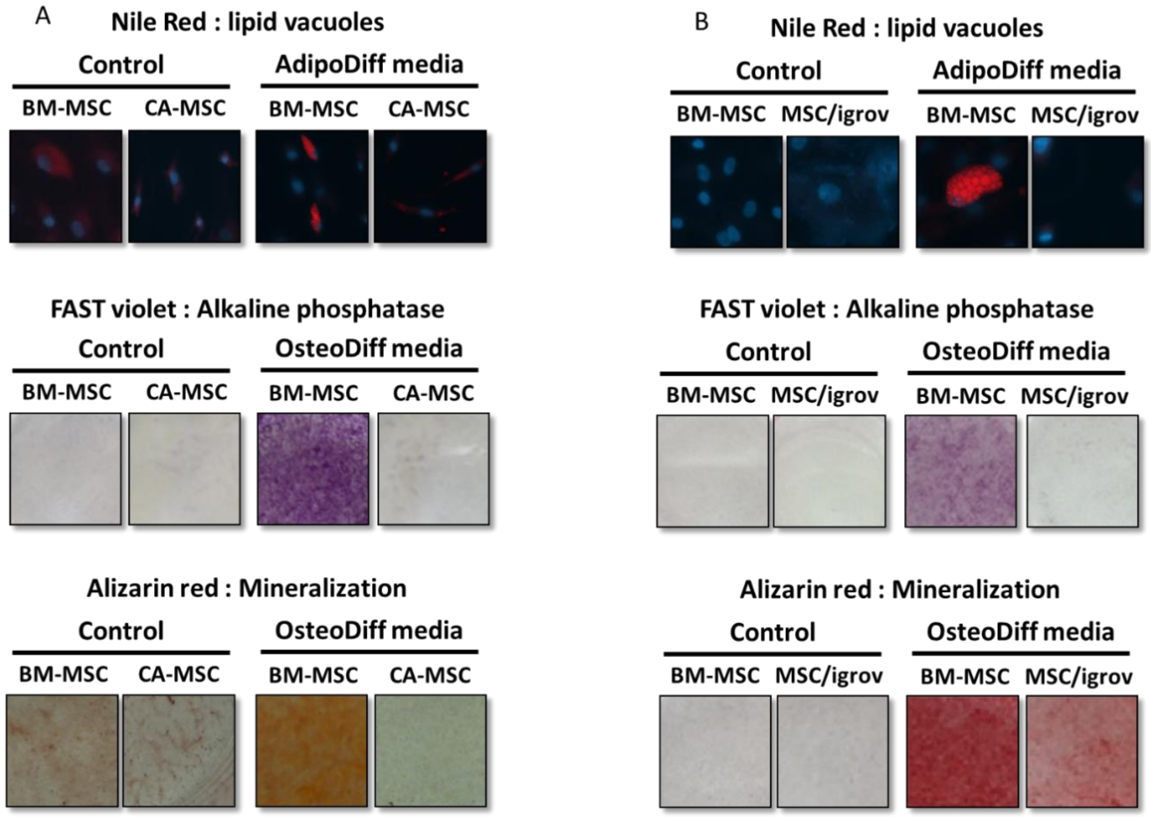

Supplementary Figure S2: Functional characterization of CA-MCS multipotency.

BM-MSCs (A and B), CA-MSCs (A) and iCA-MSCs (B) were cultured for 21 days prior to colorimetric tests. Colorimetric tests were performed using Nile red as an adipocyte stain, FAST violet and alizarin red as an osteoblast stain. BM-MSCs can differentiate into adipocytes or osteoblasts but CA-MSCs or iCA-MSCs seem to be unable to differentiate into adipocytes or osteoblasts.

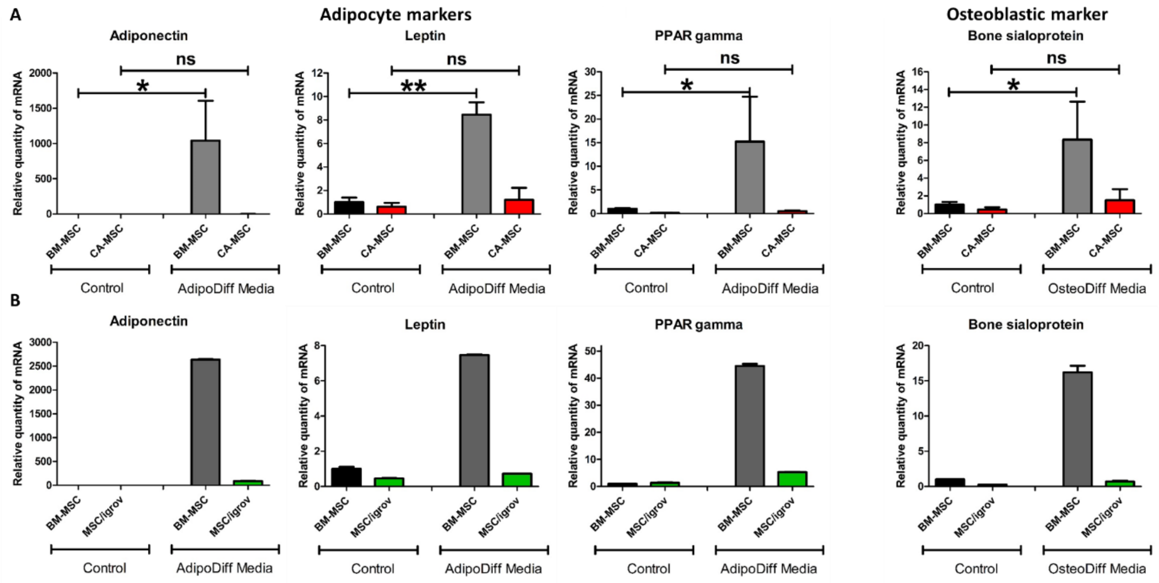

Supplementary Figure S3: Characterization of CA-MCS multipotency by transcriptional analysis.

BM-MSCs (A and B), CA-MSCs (A) and iCA-MSCs (B) were cultured for 14 days prior to RT-qPCR analysis of marker genes for adipocyte (adiponectin, leptin, PPARγ) or osteoblastic (bone sialoprotein) differentiation.

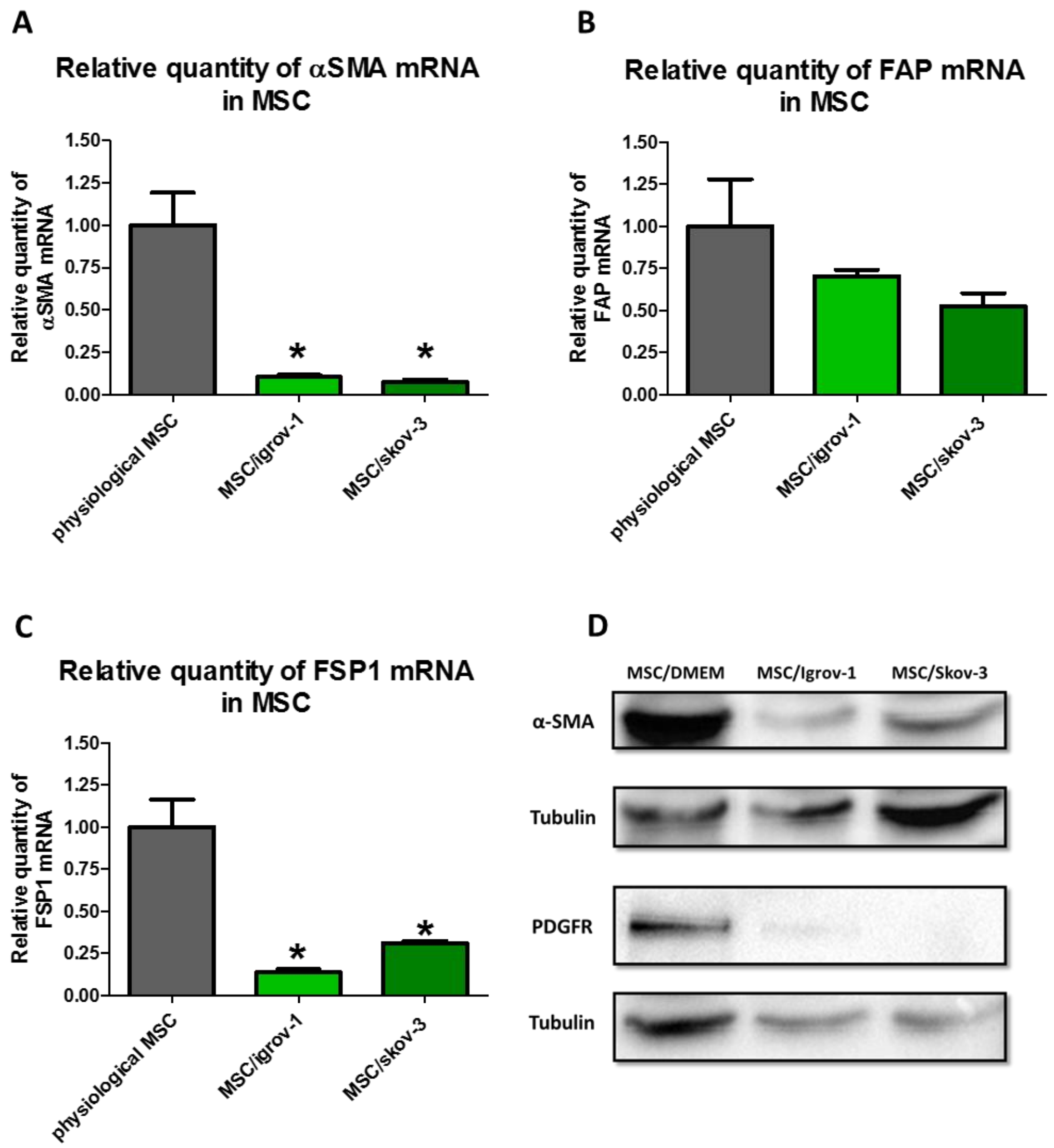

Supplementary Figure S4: Test of iCA-MSC differentiation in CAF

BM-MSCs were cultured alone or in CM from IGROV-1 (MSC/igrov-1) or SKOV-3 (MSC/skov-3) cells for 21 days. Then media were changed for complete DMEM. After three days, cells or CM were collected. A–C: The transcription of genes coding for α-SMA (A), FAP (B), FSP1 (C) was evaluated by RT-qPCR performed on mRNA extracted from BM-MSCs and different types of iCA-MSCs (induced by IGROV-1 CM or SKOV-3 CM). The data from BM-MSCs were set to 1 and the relative quantity of mRNA is shown for α-SMA (A), FAP (B), FSP1 (C). Histograms show the mean value of three independent experiments (mean +/- SEM, * $p < 0.05$). D: BM-MSCs were cultured in IGROV-1 CM, SKOV-3 CM or in control medium (physiological BM-MSCs) for 21 days. Cells were collected and proteins were extracted. PDGFR and α-SMA expression was assayed by western blot and normalized to tubulin expression (representative of 3 experiments).

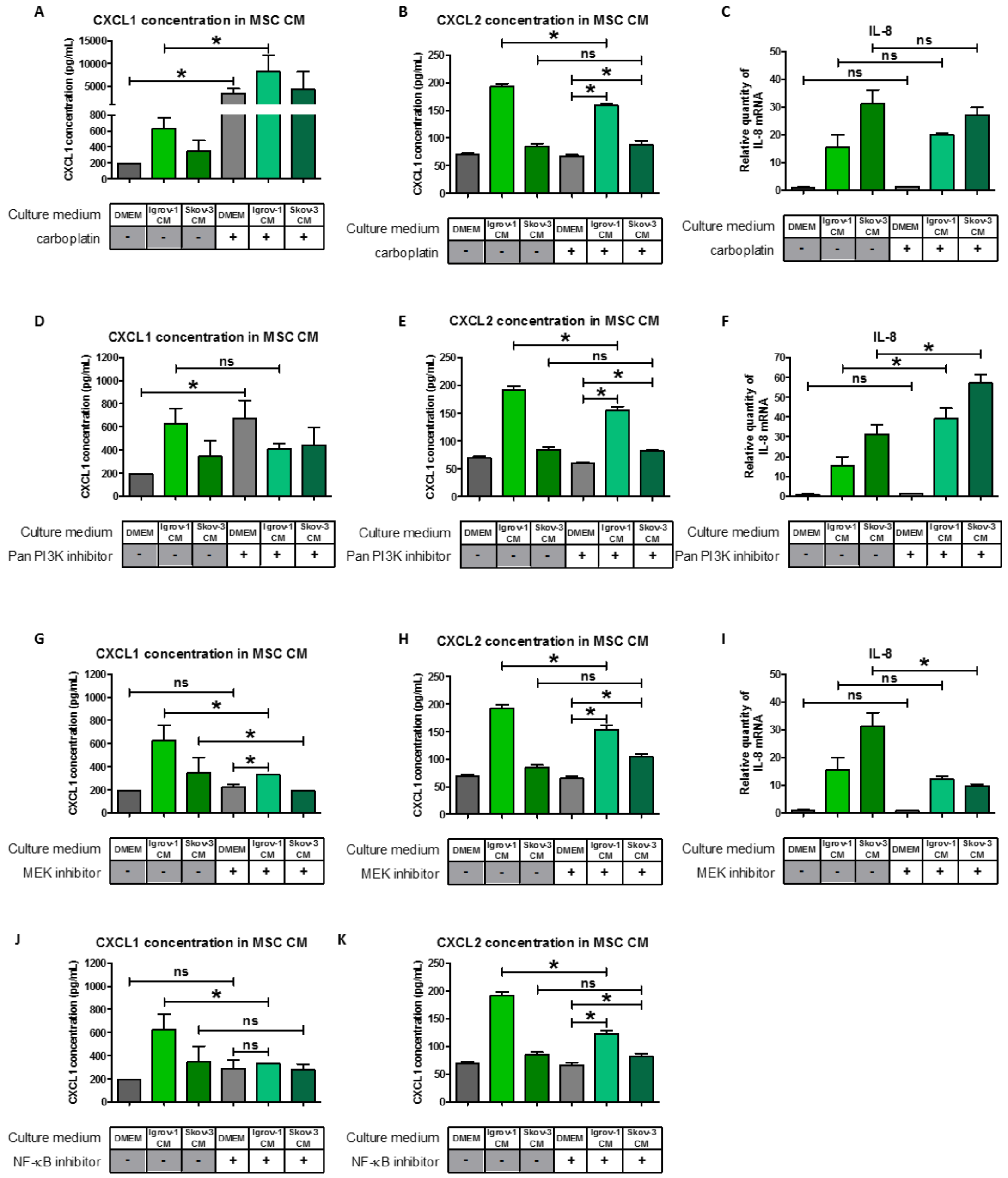

Supplementary Figure S5: Effects of treatments on the chemokine production by MSCs

BM-MSCs were cultured alone or in CM from IGROV-1 (MSC/igrov-1) or SKOV-3 (MSC/skov-3) cells for 21 days. Then media were changed for complete DMEM and cells were treated by carboplatin (50 µM) (A, B, C), a pan PI3K inhibitor, the GDC0941 (1 µM) (D, E, F), a MEK inhibitor, the AZD6244 (1 µM) (G, H, I), a NF-κB inhibitor, the Bay 11-7082 (10µM) (J, K), or vehicle (DMSO). After three days, CM or cells were collected. The concentrations of CXCL1 and CXCL2 in the CM were quantified using ELISA kits. RNA was extracted. The transcription of IL-8 gene in iCA-MSC or physiological MSC were quantified using a RT-qPCR analysis and normalized to GAPDH transcription. Histograms show the mean concentrations of three independent experiments (Mean +/- SEM, * $p < 0.05$).

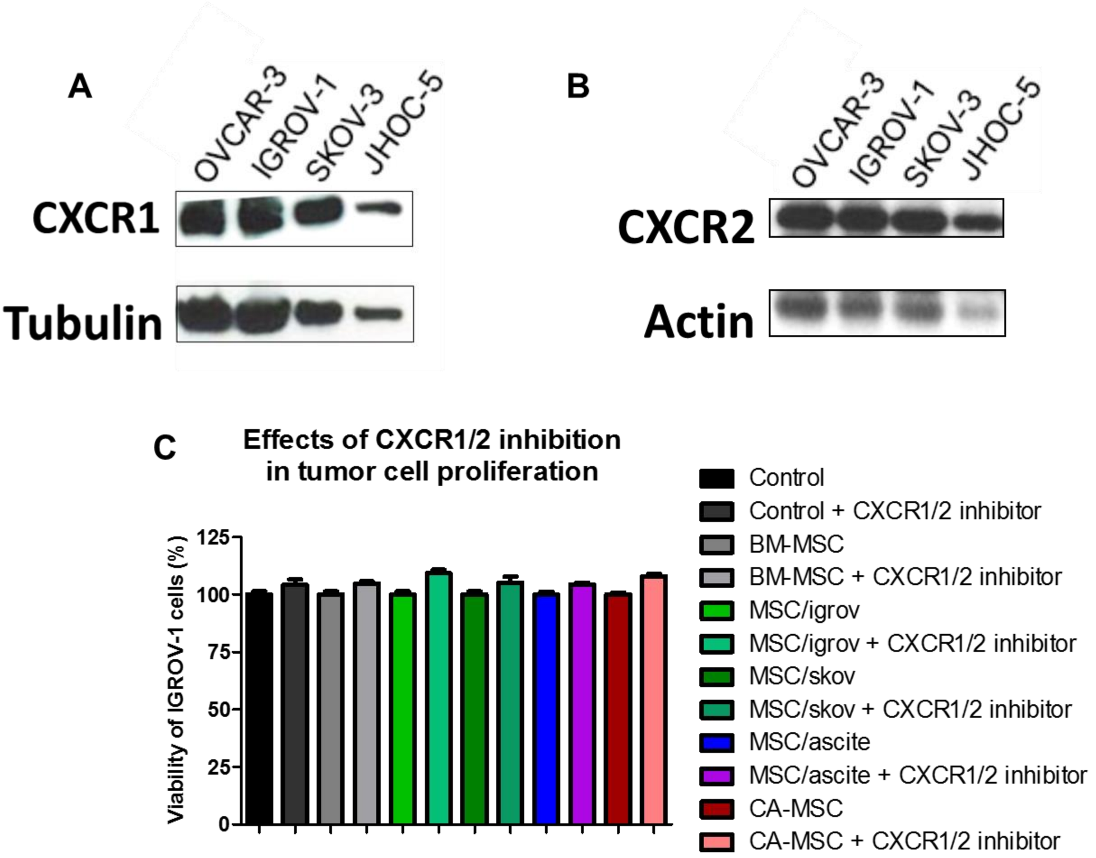

Supplementary Figure S6: Expression of CXCR1/2 and sensitivity to its inhibition in OTCs

A, B: The expression of CXCR1 (A) and CXCR2 (B) was validated by Westernblot on 4 ovarian tumor cell (OTC) lines. Tubulin and actin were used as loading control. C: Histogram representing the viability of IGROV-1 cells cultured with BM-MSC CM (physiological MSCs), CA-MSC or “induced” CA-MSC (iCA-MSC) CM from different origins (BM-MSCs cultivated with IGROV-1 CM, SKOV-3 CM or ascites) (n=4 for each type of MSC).

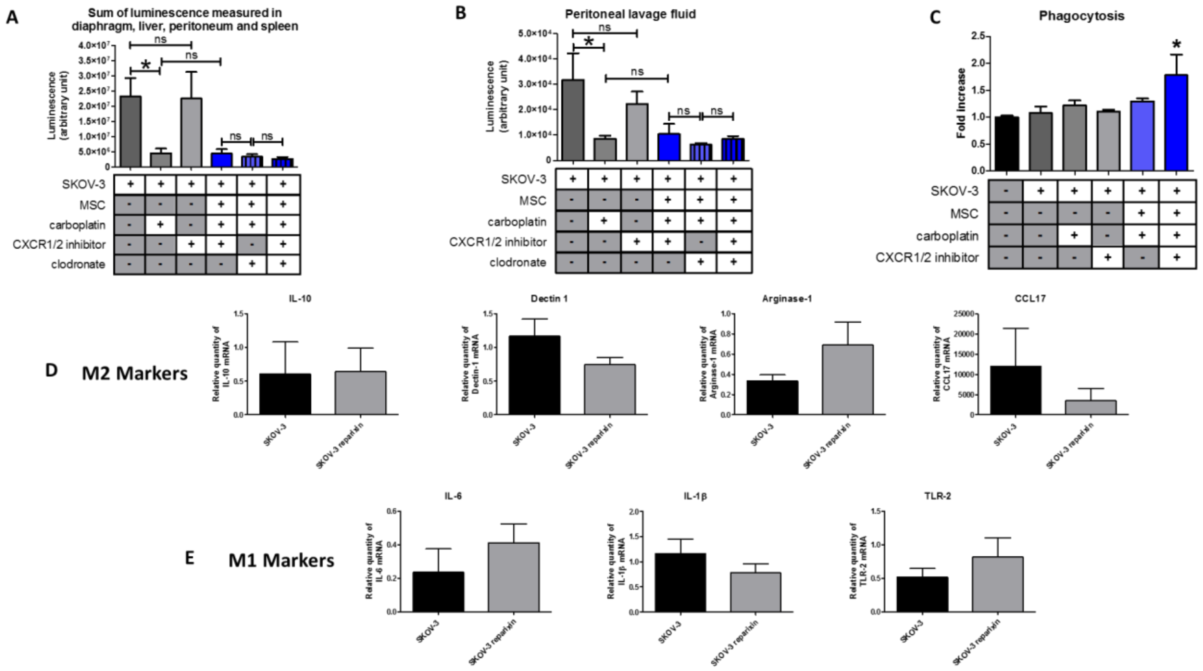

Supplementary Figure S7: Effects of the CXCR1/2 inhibitor are lost in the absence of macrophages and require their prior polarization by MSCs

A, B, C: 10^7 SKOV-3luc +/- 10^6 MSC were injected intraperitoneally (i.p.) into nude mice (6 mice/group at the beginning of the experiments). After 7 days, treatments were started, including 1 injection of carboplatin every 7 days for 3 weeks, 1 injection of reparixin 3 times per week for 3 weeks. On day 36, the mice were euthanized and a peritoneal lavage was carried out with 5 mL of NaCl 0.9%. The peritoneum, spleen, liver and diaphragm were removed +/- 1 injection of clodronate liposome once a week for 4 weeks. After addition of luciferin, the sum of the luminescence of the peritoneum, spleen, diaphragm and liver (A) and the luminescence in the peritoneal lavage fluid (B) was measured. The ability of phagocytosis of peritoneal macrophages isolated from mice was evaluated and normalized to unstimulated macrophages (C). D, E: Representative gene expression analysis of peritoneal macrophages isolated from mice that have been injected with SKOV-3 cells admixed or not with MSCs and treated with carboplatin +/- CXCR1/2 inhibitor. RNA was extracted and analyzed by RT-qPCR. The comparison between groups was performed using a Wilcoxon-Mann Whitney test (independent non-parametric data). P values of <0.05 (*) indicate a significant difference.

Supplementary Table 1: Peritoneal cancer index

Table outlining the parameters used to evaluate the peritoneal cancer index.

Maximal nodule diameter	No visible disease	< 0.5 cm	0.5 to < 2 cm	≥ 2 cm
Diameter score	0	1	5	10

Number of nodules	1-5	6-10	11-15	16-20	21-25	>25
Number score	1	2	3	4	5	6

Peritoneal cancer index = (Diameter score) x (Number score)

Supplementary Table 2: Baseline characteristics of patient samples.

Number	Type	Stage	Chemotherapy	Free interval	Sensitivity
1	**High-grade serous ovarian carcinoma**	**IIIc**	**Carboplatin/paclitaxel**	**>36 months**	**Sensitive**
2	**Ovarian carcinoma with low differentiation**	**IV**	**Carboplatin/paclitaxel**	**Progressive disease**	**Refractory**
3	**High-grade serous ovarian carcinoma**	**III**	**Carboplatin/paclitaxel**	**30 months**	**Sensitive**
4	**Serous papillary ovarian carcinoma**	**IIIc**	**Carboplatin/paclitaxel**	**5 months**	**Resistant**
5	**Serous papillary ovarian carcinoma**	**IV**	**Carboplatin/paclitaxel**	**>48 months**	**Sensitive**
6	**Clear cell carcinoma**	**IIIc**	**Carboplatin/paclitaxel**	**7 months**	**Sensitive**
7	**High-grade serous ovarian carcinoma**	**II**	**Carboplatin/paclitaxel**	**14 months**	**Sensitive**
8	**Serous papillary ovarian carcinoma**	**IIIb**	**Carboplatin/paclitaxel**	**30 months**	**Sensitive**
9	**High-grade serous ovarian carcinoma**	**IIIc**	**Carboplatin/paclitaxel**	**22 months**	**Sensitive**
10	**Serous papillary ovarian carcinoma low grade**	**III**	**Carboplatin/paclitaxel**	**>60 months**	**Sensitive**
11	**Atypical mucinous carcinoma**	**III**	**Carboplatin/paclitaxel**	**3 months**	**Resistant**
12	**High-grade serous ovarian carcinoma**	**IV**	**Carboplatin/paclitaxel**	**8 months**	**Sensitive**
13	**High-grade serous ovarian carcinoma**	**III**	**Carboplatin/paclitaxel**	**Progressive disease**	**Refractory**
14	**Serous ovarian carcinoma**	**IIIc**	**Carboplatin/paclitaxel**	**>42 months**	**Sensitive**
15	**Low-grade serous ovarian carcinoma**	**III**	**Carboplatin/paclitaxel**	**18 months**	**Sensitive**
16	**Serous ovarian carcinoma**	**III**	**Carboplatin/paclitaxel**	**4 months**	**Resistant**
17	**High-grade serous ovarian carcinoma**	**IV**	**Carboplatin/paclitaxel**	**3 months**	**Resistant**
18	**Serous ovarian carcinoma with moderate differentiation**	**ND**	**Carboplatin/paclitaxel**	**Progressive disease**	**Refractory**
19	**Serous ovarian carcinoma**	**IV**	**Carboplatin/paclitaxel**	**3 months**	**Resistant**
20	**High-grade serous ovarian carcinoma**	**III**	**Carboplatin/paclitaxel**	**5 months**	**Resistant**
21	**Ovarian adenocarcinoma with low differentiation**	**IV**	**Carboplatin/paclitaxel**	**Progressive disease**	**Refractory**

Supplementary Table 3: Effect of iCA-MSC secretions on the transcription of M2 polarization marker genes in primary human monocytes

Human monocytes were cultured for 2 days in control media, or in CM from BM-MSCs (MSC/physio CM) or in CM from different types of iCA-MSCs (induced by IGROV-1 CM (MSC/igrov-1 CM) or SKOV-3 CM (MSC/skov-3 CM). After culture, cells were washed and IL-10 mRNA expression levels were analyzed by RT-qPCR. The relative transcription of each of the evaluated genes is calculated relative to the GAPDH transcription. The genes coding for CCL17, CD36, CD163, IDO2, IL-1ra, IL-10, PGES, TGF-β and VEGF-A are analyzed. Signs +, ++ and +++ correspond respectively to an increase in transcription, relative to that of unstimulated monocytes, by a factor greater than 2, 5 and 10.

Monocytes grown in		**Physiological MSC CM**	**MSC/igrov-1 CM**	**MSC/skov-3 CM**
M2 markers	**CCL17**		++	+
	**CD36**		++	
	**CD163**		++	+
	**IDO2**		++	+
	**IL1-RA**		++	
	**IL-10**		+++	+++
	**PGES**	+	++	+
	**TGF-β**		+	
	**VEGF-A**		+	

Supplementary Table 4: Primers used for qRT-PCR analysis

Human/Mouse gene	Primer reverse	Primer forward	Species
**α-SMA**	ACTGGGACGACATGGAAAAG	TACATGGCTGGGACATTGAA	Human
**Arginase-1**	GTGAAGAACCCACGGTCTGT	CTGGTTGTCAGGGGAGTGTT	Mouse
**CCL17**	AGGTCTTGAAGCCTCCTCAC	AGTTCAGACAAGGGGATGGG	Human
**CCL17**	AGTGGAGTCTTCCAGGGATG	CTGGTCACAGGCCGTTTTAT	Mouse
**CD36**	GGTGTGGTGATGTTTGTTGC	CAGGGCCTAGGATTTGTTGA	Human
**CD163**	AAGCTGATGTGGTTTGCAGG	CCATTGCCAGTTCTTGCAGT	Human
**CXCL1**	AGGGAATTCACCCCAAGAAC	CACCAGTGAGCTTCCTCCTC	Human
**CXCL2**	CGCCCAAACCGAAGTCATAG	AGACAAGCTTTCTGCCCATTCT	Human
**Dectin-1**	CATCGTCTCACCGTATTAATGCAT	CCCAGAACCATGGCCCTT	Mouse
**FAP**	CCAGGAGATCCACCTTTTCA	ACGCAGGGTAAGTGGTATCG	Human
**FSP1**	GATGAGCAACTTGGACAGCA	CCCAACCACATCAGAGGAGT	Human
**GAPDH**	AGGTCGGAGTCAACGGATTT	ATCTCGCTCCTGGAAGATGG	Human
**GAPDH**	AACTTTGGCATTGTGGAAGG	ACACATTGGGGGTAGGAACA	Mouse
**IDO2**	CTGGTCCTGAGCTTCCTCAC	CAGCACCAAGTCTGAGTGGA	Human
**IL-1β**	GATCCACACTCTCCAGCTGCA	CAACCAACAAGTGATATTCTCGATG	Mouse
**IL-1ra**	TGGGAATCTCAGATGGGAAG	CTGTGTCCCCCAGAACTTGT	Human
**IL-6**	TACCCCCAGGAGAAGATTCC	TTTTCTGCCAGTGCCTCTTT	Mouse
**IL-8**	ACACTGCGCCAACACAGAAATTA	TTTGCTTGAAGTTTCACTGGTATC	Human
**IL-10**	TGCAAAACCAAACCACAAGA	TCTCGGAGATCTCGAAGCAT	Human
**IL-10**	CCAAGCCTTATCGGAAATGA	TTTTCACAGGGGAGAAATCG	Mouse
**PGES**	CATGTGAGTCCCTGTGATGG	GACTGCAGCAAAGACATCCA	Human
**TGF-β**	ACTGAGGGGAAGGGACAACT	TCGGTACCAGGTGAGGGTAG	Human
**VEGF**	CTTCTGAGTTGCCCAGGAGA	CTCACACACACACAACCAGG	Human
